# Instrumental music teachers’ perceptions and acceptance of Al integration in teaching: a mixed-methods study based on the UTAUT2 model

**DOI:** 10.3389/fpsyg.2026.1887153

**Published:** 2026-07-08

**Authors:** Yijie Zhang, Shuhui Niu

**Affiliations:** 1 Moscow State Normal University, Moscow, Russia; 2 Luoyang Normal University, Luoyang, China

**Keywords:** artificial intelligence, instrumental music education, mixed methods, technology acceptance, UTAUT2

## Abstract

**Introduction:**

The application of Artificial Intelligence (AI) is fast becoming widespread in educational environments, especially within the field of music education. However, instrumental music teachers, who are highly dependent on their physical instructions and aesthetic judgement, have never undergone an analysis of how they perceive and embrace AI technologies. This study is based on an expanded version of Acceptance and Use of Technology (UTAUT2), which includes two additional variables that relate to the context: Perceived Threat to Teaching Artistry (PTTA) and Perceived Irreplaceability of Embodied Teaching (PIET).

**Methods:**

The method employed by this study was an explanatory sequential mixed methods approach, wherein the first phase involved the use of Partial Least Squares Structural Equation Modeling (PLS-SEM) on survey data gathered from 352 in-service instrumental music teachers in China. In the qualitative phase, reflexive thematic analysis was conducted on semi-structured interviews with 17 instrumental music teachers.

**Results:**

Results show that performance expectancy, habit, hedonic motivation, effort expectancy, and social influence significantly and positively predict behavioral intention, while facilitating conditions and price value show no significant effect. Both PTTA and PIET significantly and negatively affect behavioral intention, with PIET’s negative effect being more pronounced among experienced teachers and string/wind instrument teachers. The qualitative phase yielded four core themes, revealing a conditional acceptance pattern: teachers acknowledge AI’s supplementary value in basic skill training but firmly maintain their irreplaceable role in aesthetic judgment, individualized expressive guidance, and embodied interaction.

**Discussion:**

This study extends UTAUT2’s applicability to the instrumental music teaching context, where artistry and embodiment are central, providing empirical evidence for designing and promoting AI teaching tools.

## 1 Introduction

The adoption of AI technology in education is at an all-time high. There are numerous applications of the interrelationship between technology and instruction that are revolutionizing the educational ecosystem. The same trend can be seen in music education, where there are many innovative tools utilizing AI technology in tasks such as practice accompaniment, pitch/rhythm evaluation, and arrangement guidance (Yu et al., 2023). In addition, empirical studies have found that the integration of AI-based instruments into instrumental learning environments, like piano classes, can enhance learners’ achievements and motivation to a certain degree (Li and Wang, 2024). However, simply having access to such instruments does not necessarily mean that teachers will use them; their perspectives on the introduction of new technology require further study.

In the literature on technology acceptance in education, the views and attitudes of teachers are well-recognized as significant influencing factors for the successful integration of technology. However, studies show that although teachers perceive the role of AI-based technologies in improving student learning, their use is still limited due to various reasons, such as low digital literacy among teachers (Yim and Wegerif, 2024). Where the implementation of new technology raises the question of identity within the profession, teachers are likely to have more complicated psychological reactions—while there is a common agreement that AI cannot substitute for the uniquely human features that make teachers indispensable when cultivating the general skills of their students, this understanding does not eliminate teachers’ fears of marginalization of their roles completely (Chan and Tsi, 2024). The inconsistency may become particularly relevant in academic fields where the expectation of significant interaction with learners is present.

Instrumental music teaching provides an example of such a situation, particularly when it occurs at an advanced level. From a technological standpoint, technology for the study of musical instruments has developed through many years of progress, moving from hardware to mobile devices, encompassing areas such as pitch, rhythm, finger placement, and performance metrics. However, such technology is far from being capable of addressing complex areas like tone evaluation and nuanced assessment of performance technique (Acquilino and Scavone, 2022). Most importantly, instrumental teaching has its own disciplinary features: It requires extensive interaction between teacher and learner through embodied guidance related to proper placement, breathing, and posture, along with other artistic skills of teaching like aesthetic appreciation and musical performance, which cannot be algorithmically determined. The above factors indicate the possible differences in perception and acceptance of AI among instrumental teachers compared to teachers in other subject domains, although existing studies on technology acceptance have overlooked these teachers.

Theoretically speaking, UTAUT2, which is a very popular framework for examining technology adoption, has been frequently used in educational technology acceptance research. It has been systematically reviewed and theoretically analyzed that the model demonstrates good reliability in most aspects, and context-based extension of models has been identified as one of the most prevalent research trends (Tamilmani et al., 2021b). There have been attempts to explore the UTAUT2 framework for the music education context; however, such research was restricted to preservice music teachers who did not have actual teaching experience and followed a purely quantitative approach, without taking into consideration variables unique to the instrumental music teaching context (He and Ren, 2025). This indicates that, while exploring the phenomenon of AI impact on teaching practice among instrumental music teachers who actually work with AI, there is no theoretical basis for explaining their acceptance process.

In light of these identified gaps, this study intends to extend the UTAUT2 framework by incorporating two context-specific variables (PTTA & PIET) to develop a technology acceptance model applicable to the instrumental teaching environment. The study will adopt an explanatory sequential mixed method approach to analyze the following research questions: (RQ1) How do the UTAUT2 variables along with the context-specific variables affect the behavioral intention and use behavior of the instrumental teachers towards AI teaching tools? (RQ2) What do the instrumental teachers perceive about the value, threat, and limitations of AI in the context of instrumental teaching, and how is this perception related to important UTAUT2 variables?

This contribution can be explained from both practical and theoretical perspectives. Theoretical contribution includes the extension of the usefulness of UTAUT2 into the domain of instrumental teaching that heavily relies on bodily interactions and artistic skills. With the proposal and verification of the proposed models, i.e., PTTA and PIET, this work expands the potential of technology acceptance theory to consider the aspects related to disciplines. In terms of practice, this work contributes to the empirical knowledge base for designing and implementing AI-based tools for instrumental teaching.

## 2 Literature review

### 2.1 Current applications of AI in music education

AI applications for music education have developed into a varied range of tools. They are reviewed systematically in four categories that include intelligent tutors, composition aid, performance assessment, and adaptive learning systems (Merchán Sánchez-Jara et al., 2024). When it comes to assessment of the students’ progress, AI has great potential to provide immediate and accurate feedback regarding their performance, thus contributing to personalized instruction. But at the same time, AI poses an important challenge to current assessment and grading approaches (Shaw, 2024). The generative AI tool allows students to create music using only text-based prompts, yet at the same time, there is a need for caution regarding the issue of cultural bias, the definition of originality, equality in education, and ethical aspects (Cheng, 2025a). Concerning educational administration, there is evidence that suggests Artificial Intelligence (AI) holds much promise in assessing instruction and resource allocation. However, there are still limitations to current approaches, particularly in their ability to extrapolate information into different settings and adjust to varying conditions within music education systems (Wu, 2025).

### 2.2 Evolution of technology acceptance theory and the UTAUT2 model

UTAUT2 extends UTAUT with three consumer-oriented factors—hedonic motivation, price value, and habit—forming a seven-construct model (Venkatesh et al., 2012). Systematic reviews confirm its continued effectiveness in educational technology contexts, though construct significance varies across settings (Xue et al., 2024), and meta-analytic evidence supports the model’s overall robustness while identifying context-dependent extension as the dominant research trend (Tamilmani et al., 2021a).

For specific instances of educational technology usage, an empirical investigation on the intention to adopt AI tools by Chinese higher education teachers reveals that effort expectancy is the most influential determinant of intention, while habit is the most significant influence on usage behavior. Moderation effects of gender, age, and experience were statistically insignificant (Xu et al., 2024). Regarding the use intention continuity of virtual reality technology by teachers, UTAUT2 showed a high level of explanatory ability, thus stressing the necessity to pay attention to the facilitating condition and performance expectancy (Du and Liang, 2024). The abovementioned works prove that UTAUT2’s explanatory ability is strongly related to the proper adaptation to particular environments, and the high specificity of instrumental teaching becomes such an environment.

### 2.3 Disciplinary specificity of instrumental teaching and technology acceptance

The unique character of instrumental learning can be described through the importance of embodiment in the process of teaching and learning. Music teaching that is based on embodiment highlights the idea that both the physical and expressive processes involved in learners and teachers become central in the process of music instruction; there is an essential connection between movement and sound (Bremmer and Nijs, 2022). Performance instruction at the micro level is heavily influenced by performers’ awareness and control of their internal physical states, such as muscle tension, breath regulation, and body posture. In other words, a substantial portion of knowledge in instrumental instruction is embodied knowledge; the teaching process involves transferring tacit knowledge through touch, imitation, and postural adjustment (Alves and Nogueira, 2024).

This inherent reliance on embodied interaction poses special difficulties for the implementation of technologies within the domain. Instrumental teachers have been shown through qualitative studies to recognize the usefulness of the technologies as secondary aids to the training process while recognizing that their non-integration into the daily training process is due to the lack of pedagogic focus in technology design (Michałko et al., 2022). Analysis of empirical data on piano tone color instruction indicates the complicated mechanism of transferring knowledge of tonal understanding through verbal explanation, performance illustration, touch, and guidance—an instructional strategy that involves many modes of interaction and relies greatly on immediate decision-making processes that are hard to imitate by technology (Li and Timmers, 2021).

From the above analysis, it is possible to determine the two main factors which determine the specificity of instrumental instruction, both of which are directly linked to technology adoption. The first factor involves the artistry aspect, where the instructor must help the learner develop their sense of judgement as well as musical expressiveness, tasks that depend on personal development by the teacher. With regard to embodiment, teaching tasks involving correcting hand placement, controlling breathing, and adjusting posture involve tactile interaction, which is difficult for present AI technology to handle. As a result, the following context-based variables are proposed in this paper: Perceived Threat to Teaching Artistry (PTTA), indicating teachers’ fear of being threatened by AI’s standardized evaluation of their artistic ability to teach; and Perceived Irreplaceability of Embodied Teaching (PIET), indicating teachers’ perception that hands-on, physical instruction cannot be substituted for by AI.

### 2.4 Conceptual framework and research hypotheses

**Figure 1:**
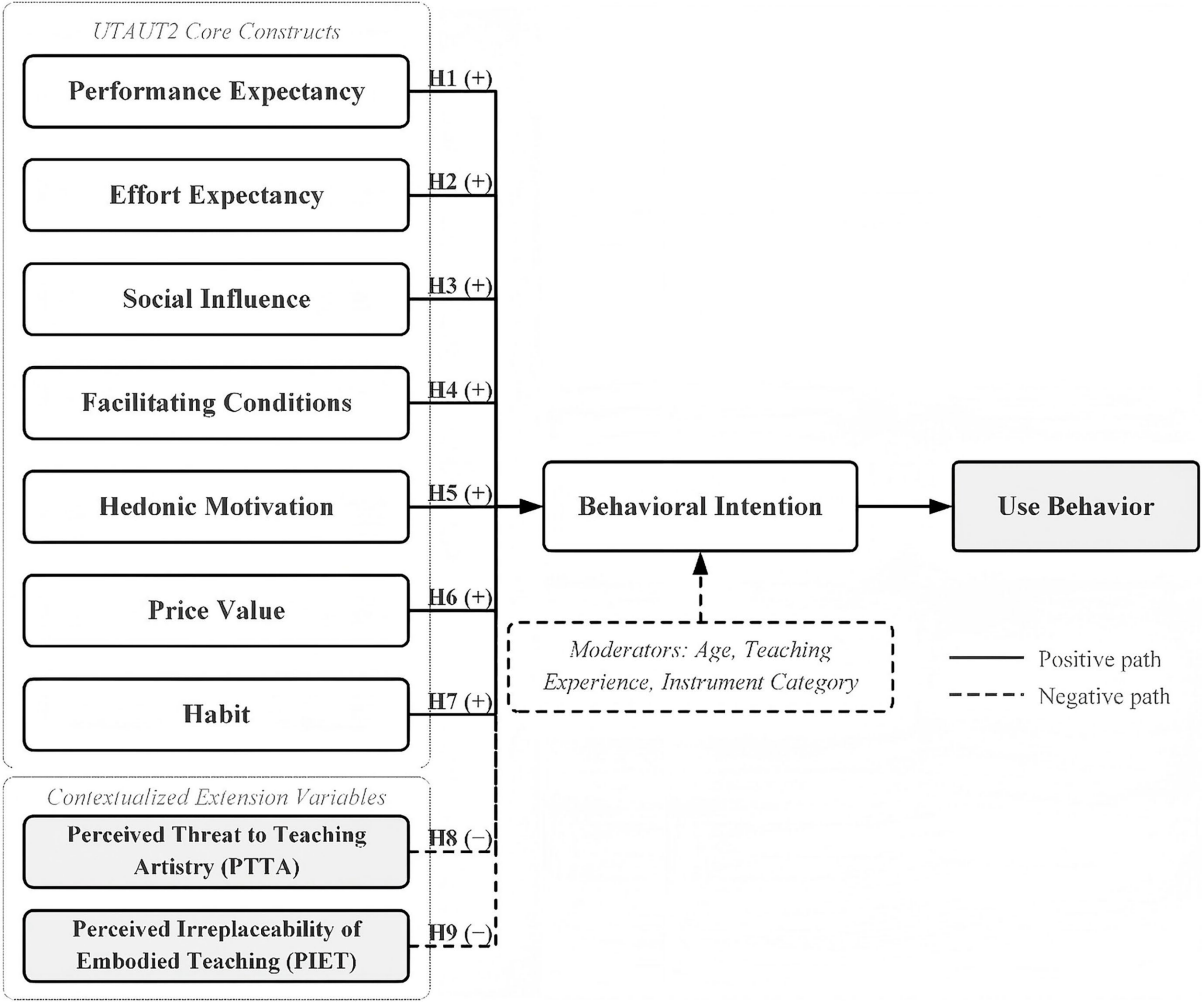
Proposed research model: extended UTAUT2 for instrumental music teaching context.

Integrating UTAUT2 core theory with the analysis of instrumental teaching’s disciplinary specificity, this study constructs an extended UTAUT2 conceptual model (see Figure 1). The model includes UTAUT2’s seven original constructs plus PTTA and PIET, all predicting behavioral intention as the core endogenous variable, which in turn predicts use behavior.

In relation to the original paths in UTAUT2, the performance expectancy denotes how much teachers anticipate that the use of AI tools will enhance teaching effectiveness and efficiency. Several studies have found that teachers’ acceptance of AI technologies is positively affected by their belief that AI software can actually help with teaching-related activities (Acikgul and Sad, 2026). Therefore, H1 Hypothesis is developed: positive correlation between performance expectancy and behavioral intention exists. Social influence refers to the impact of external reference groups (colleagues, administrators, and professional communities) on teachers’ adoption behavior. From ethical perception, views of educators concerning AI technology are affected by social norms and peer influence (Zhu W. et al., 2025). Thus, Hypothesis H3 states that social influence positively influences behavioral intention. Other factors suggested by the UTAUT2 model to positively influence behavioral intention are effort expectancy, facilitating conditions, hedonic motivation, price value, and habit. Thus, the hypotheses of effort expectancy (H2), facilitating conditions (H4), hedonic motivation (H5), price value (H6), and habit (H7) on behavioral intention can be stated.

As for the context-based extension of research, there is an important finding in studies related to teachers’ resistance to AI: over 35% of university professors do not use generative AI on purpose due to concerns about the degradation of human intellect (Shata, 2025). Potential threat posed by AI to instrumental music educators might become even more evident considering the construct of teaching artistry since educators who fear that AI’s objective feedback might compromise their ability to discern and express themselves aesthetically will have less inclination to embrace it. Consequently, Hypothesis H8 states: PTTA negatively impacts behavioral intention. Regarding the line of study on technology acceptance of music education, it is clear that the former literature has utilized contextual factors such as perceived compatibility in the UTAUT2 model and validated their usefulness (Chen et al., 2026), making it feasible to employ domain-specific factors in the case of instrumental lessons. Regarding the embodiment teaching aspect, if the teachers have strong beliefs about the irreplaceability of the embodied teaching activities by AI due to current technological limitations, this irreplaceability belief may undermine their willingness to investigate AI technologies. Studies have proven that personal factors like anxiety and perceived risk act as barriers in adopting educational technology (Caffaratti et al., 2025). Therefore, we put forward Hypothesis H9: PIET negatively influences behavioral intention.

With regard to moderating factors, there are three: age, experience as a teacher, and instrument category. Age is a critical factor since teachers of various ages have varying levels of digital literacy as well as willingness to accept technology. The latter could mean that the effect size of the relationship between independent variables and behavioral intention will be moderated. Years of experience may contribute to path dependence, meaning that it can affect the strength of the relationship for PTTA and PIET.

## 3 Methodology

### 3.1 Research design overview

**Figure 2:**
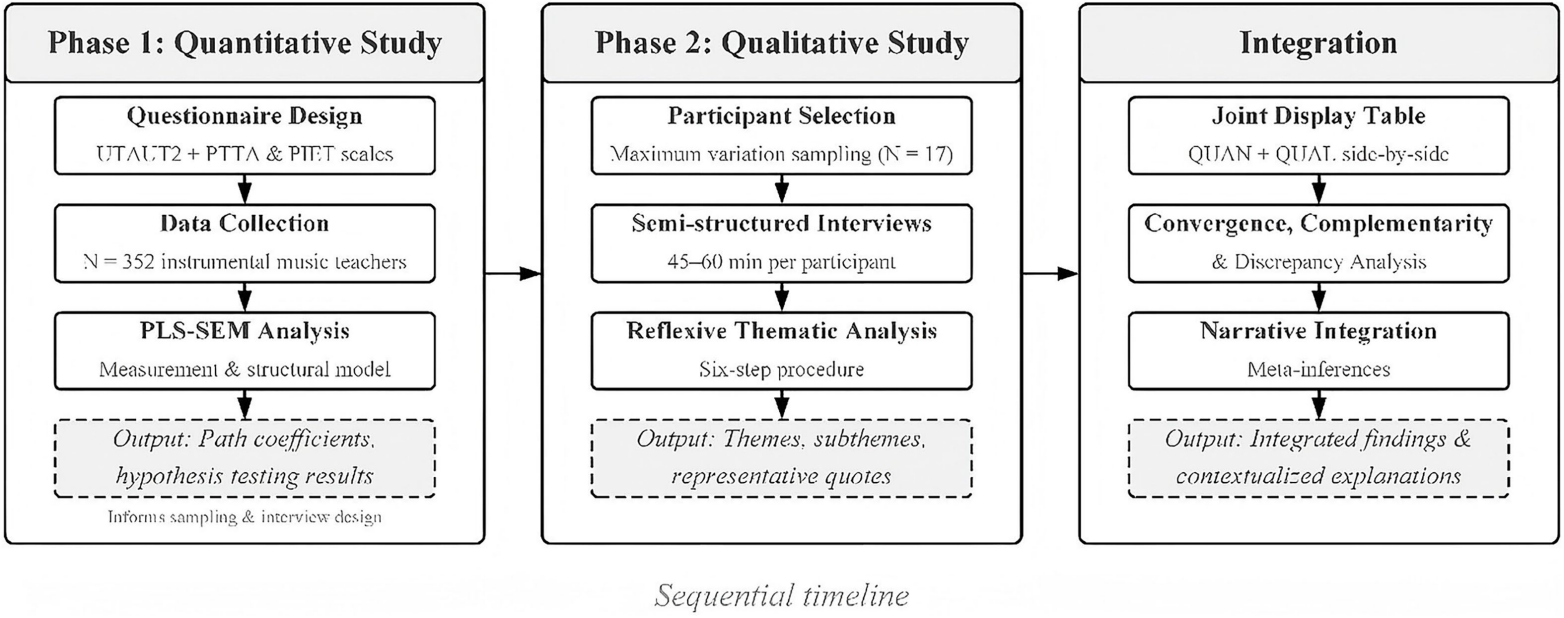
Research design: explanatory sequential mixed-methods procedure.

**Figure 3:**
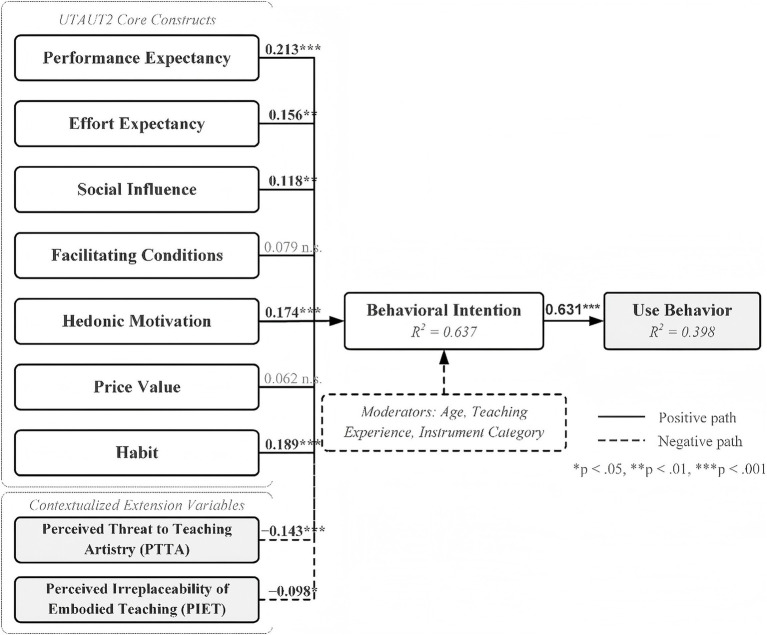
Structural model results with standardized path coefficients.

This study employed an explanatory sequential mixed-methods design (Creswell and Plano Clark, 2023; Creswell and Creswell, 2017), proceeding in two phases (see Figure 2). Phase 1 tested the extended UTAUT2 model through a survey analyzed with PLS-SEM. Phase 2 used semi-structured interviews, with participants selected on the basis of Phase 1 results, to explain significant and non-significant paths through reflexive thematic analysis (see Figure 3).

### 3.2 Phase 1: quantitative study

The survey was developed using the original UTAUT2 measures, with the wording being altered according to its relevance within an instrumental teaching setting. Items relating to the seven constructs of performance expectancy, effort expectancy, social influence, facilitating conditions, hedonic motivation, price value, and habit were derived from the original UTAUT2 measure, which consisted of 3–4 items per construct and was rated on a 7-point Likert scale. PTTA and PIET are newly developed constructs with no directly adoptable scales in the existing literature. Their item development followed a three-stage procedure. In the first stage, an initial pool of eight candidate items per construct was generated deductively from the theoretical analysis presented in Section 2.3: PTTA items captured concerns about AI’s standardized evaluation undermining personalized artistic judgment, while PIET items addressed beliefs that tactile correction, postural guidance, and breath demonstration cannot be replicated by current AI technology. In the second stage, a panel of five subject-matter experts—three in instrumental pedagogy and two in educational technology—evaluated item relevance and clarity. Items with an item-level content validity index (I-CVI) below 0.80 were revised or eliminated, resulting in four items retained for each construct. In the third stage, cognitive interviews were conducted with six practicing instrumental teachers to verify comprehension and response process validity; minor wording adjustments were made based on their feedback. The final measurement items for PTTA and PIET each comprised three items after one additional item per construct was removed during the subsequent pilot test due to redundancy identified through inter-item correlation analysis. The translation followed a rigorous forward–backward procedure. Two bilingual researchers independently translated the English-language UTAUT2 items into Chinese; discrepancies were reconciled through discussion with a third bilingual researcher holding expertise in music education. A separate translator, blind to the original version, back-translated the Chinese version into English. The back-translated version was compared with the source instrument, and discrepancies were resolved by the research team to ensure semantic, idiomatic, and conceptual equivalence. For the qualitative phase, all interviews were conducted in Chinese. Transcripts were analyzed in the original Chinese to preserve contextual nuance, and only the representative quotes presented in the manuscript were translated into English by a bilingual member of the research team and verified by an independent reviewer. The initial survey included fifty instrumental music teachers for the pilot test. An exploratory factor analysis validated that all of the items fell on the respective construct domains. Initial reliability tests resulted in Cronbach’s alpha coefficients ranging from 0.78 to 0.91. The UB construct domain had one item dropped for high cross-loading, and all other items passed the psychometric test.

Sampling-wise, the target population is described as practicing teachers who are involved in instrumental music teaching within different music education institutes in China. This covers instructional courses offered in piano, strings, winds, and percussion instrument areas. Purposive sampling with the help of snowball sampling was used for gathering information from various sources, such as professional associations of music educators, conservatories, and institutions providing instrumental music courses. Sample size calculation was done using the power analysis approach using G*Power software. At the effect size *f*^2^ = 0.05 (small), significance level *α* = 0.05, power 1 − *β* = 0.80, and 9 predictors, the sample size needed was around 277 individuals. To allow for several groupings and non-validation of data, the research decided to have more than 350 participants (Hair et al., 2021).

Data were collected using an online survey tool over the period of 6 weeks. The quality control process involved the use of two attention-check questions (responses failing these were excluded), response time criterion (under 3 min deemed invalid), and IP address deduplication to eliminate repeated submissions.

PLS-SEM was selected over covariance-based SEM (CB-SEM) for three methodological reasons. PTTA and PIET are newly developed constructs without well-established nomological networks, and PLS-SEM is recommended over CB-SEM in such exploratory contexts where theory is being extended rather than confirmed (Hair and Alamer, 2022). In addition, this study prioritizes maximizing the explained variance of the two endogenous variables (behavioral intention and use behavior), which aligns with PLS-SEM’s prediction-oriented objective rather than CB-SEM’s emphasis on model fit and parameter recovery. Assessment of univariate skewness and kurtosis further indicated that several indicators deviated from normality, and PLS-SEM’s distribution-free estimation is better suited to such conditions. SmartPLS 4.0 was used, following a two-step procedure: measurement model assessment examined internal consistency reliability (Cronbach’s *α* and composite reliability), convergent validity (average variance extracted, AVE), and discriminant validity (Fornell–Larcker criterion and HTMT ratio); structural model assessment examined path coefficient significance, *R*^2^ values, effect sizes *f*^2^, and predictive relevance *Q*^2^. A systematic review of PLS-SEM applications in educational technology research over the past decade further confirms this method’s suitability for the present study (Demir and Uşak, 2025).

### 3.3 Phase 2: qualitative study

Interviewees were selected from Phase 1 respondents through maximum variation sampling guided by three stratification criteria. First, respondents were classified into high, medium, and low AI acceptance groups based on their mean behavioral intention scores (top quartile, interquartile range, and bottom quartile, respectively). Within each acceptance group, candidates were further stratified by instrument category (piano, strings, winds, percussion) and teaching experience (≤10 years vs. >10 years) to maximize diversity across these theoretically relevant dimensions. Invitations were sent to candidates in each cell until saturation of the sampling matrix was approached, yielding a final sample of 17 participants whose profiles are detailed in Table 1.

**Table 1:** Interview participant profiles (*N* = 17).

ID	Gender	Age	Instrument	Experience (yrs)	Institution type	AI acceptance level
T1	F	29	Piano	5	Private school	High
T2	M	42	Violin	18	Conservatory	Low
T3	F	34	Flute	9	University	Medium
T4	M	51	Cello	26	Conservatory	Low
T5	F	27	Piano	3	Private school	High
T6	F	38	Clarinet	14	University	Medium
T7	M	45	Percussion	20	Conservatory	Medium
T8	F	31	Piano	7	Private school	High
T9	M	36	Violin	12	University	Low
T10	F	47	Erhu	22	Conservatory	Low
T11	M	33	Trumpet	8	University	Medium
T12	F	40	Piano	16	Conservatory	Medium
T13	F	30	Guitar	6	Private school	High
T14	M	53	Viola	28	Conservatory	Low
T15	F	35	Flute	11	University	Medium
T16	M	26	Percussion	2	Private school	High
T17	F	44	Piano	19	University	Low

For the interviews that utilized a semi-structured approach, the design had to consider two aspects: addressing the various themes related to each construct under the UTAUT2 framework (such as teachers’ perception of the usefulness of the AI applications in teaching, challenges of using the technology, attitudes of peers and institutions towards the adoption of technology). The probing questions for both PTTA and PIET were specifically developed (e.g., “To what degree do you think AI is able to comprehend and evaluate students’ musical expressivity?” “How do you think the technology tools may assist in hand positioning and posture correction?”). The interviews took about 45–60 min, and all interviews were audio-taped with the respondents’ permission.

Qualitative data analysis was conducted through reflexive thematic analysis, based on the six steps traditionally employed for such a process, including data familiarization, initial coding, theme identification, theme refinement, theme definition, and report writing (Braun and Clarke, 2021b). The coder remained aware of his theoretical preconceptions during the coding process, ensuring that he did not force the UTAUT2 model upon the qualitative data. In order to ensure quality control, the present research adopted rigorous practices regarding reflexive thematic analysis in which coding logs and reflective memos were meticulously kept while critical discussion among researchers ensured that the generated themes had both internal homogeneity and external heterogeneity (Braun and Clarke, 2021a).

### 3.4 Integration strategy

In this paper, the joint display method was adopted as the main method for integrating qualitative and quantitative data through juxtaposition of quantitative path coefficients and hypothesis testing results along with the relevant themes in qualitative data (Fetters and Tajima, 2022). There were three possible cases for data integration: first, qualitative data explaining the context and mechanism of statistically significant paths; second, qualitative data examining possible moderating or suppressing conditions of statistically insignificant paths; third, qualitative data discovering other variables and relationships not covered in quantitative models.

### 3.5 Ethical considerations

This study received approval from the institutional ethics review board. All participants signed informed consent forms before participation, clearly stating the research purpose, data use, anonymization measures, and participant rights. The survey responses and audio recordings from interviews were all made anonymous, and the participants were assigned codes (such as T1 and T2) instead of identifiable information. The raw data collected is kept on secured servers, which can be accessed by the research team. Participants could withdraw at any time without adverse consequences.

## 4 Results

### 4.1 Phase 1: quantitative results

A total of 520 surveys were distributed using the online survey platform, with 387 surveys successfully retrieved (valid response rate = 74.4%). Participants who did not pass both attention checks (21), participants who completed the survey within 3 min (9), and participants with duplicate IPs (5) were disqualified. As such, 352 surveys were kept as valid (validity rate = 90.9%). The demographic data for our sample are presented in Table 2.

**Table 2:** Demographic characteristics of survey respondents (*N* = 352).

Variable	Category	*n*	%
Gender	Male	118	33.5
	Female	234	66.5
Age	25 and below	47	13.4
	26–35	139	39.5
	36–45	108	30.7
	46 and above	58	16.5
Teaching experience	≤5 years	89	25.3
	6–10 years	112	31.8
	11–20 years	98	27.8
	>20 years	53	15.1
Instrument category	Piano	126	35.8
	Strings	87	24.7
	Winds	78	22.2
	Percussion	37	10.5
	Other	24	6.8
Institution type	Music conservatory	94	26.7
	University music department	121	34.4
	Private music school	107	30.4
	Other	30	8.5

The majority of the sample were females (66.5%), mirroring the actual gender distribution in the target group population. Age and experience distributions appeared similar for the selected participants, with the 26–35 and 6–10 years categories representing the largest groups, implying that the sample was mainly composed of early-to-mid career teachers while also covering different career stages. When categorized according to the type of instrument taught, piano teachers made up the majority of the sample (35.8%) while string teachers came in second (24.7%), followed by wind instruments (22.2%) and finally percussion instruments (10.5%). The results reflect the true difference in scale between the different types of instruments taught in China.

Measurement model assessment results are presented in Table 3. In the initial analysis, UB4 had a factor loading of 0.587, below the 0.70 threshold, and was deleted before re-estimating the model. All retained items had factor loadings ranging from 0.714 to 0.923, exceeding the 0.70 standard. Cronbach’s *α* values ranged from 0.782 to 0.912 and composite reliability (CR) values from 0.873 to 0.938, all exceeding the 0.70 threshold, indicating good internal consistency reliability. AVE values ranged from 0.581 to 0.795, all exceeding the 0.50 threshold, confirming adequate convergent validity.

**Table 3:** Measurement model results: factor loadings, reliability, and convergent validity.

Construct	Item	Loading	Cronbach’s α	CR	AVE
Performance expectancy (PE)	PE1	0.867	0.872	0.921	0.795
	PE2	0.903			
	PE3	0.904			
Effort expectancy (EE)	EE1	0.846	0.838	0.903	0.756
	EE2	0.879			
	EE3	0.883			
Social influence (SI)	SI1	0.832	0.812	0.889	0.727
	SI2	0.871			
	SI3	0.854			
Facilitating conditions (FC)	FC1	0.793	0.782	0.873	0.632
	FC2	0.812			
	FC3	0.786			
	FC4	0.789			
Hedonic motivation (HM)	HM1	0.891	0.867	0.918	0.789
	HM2	0.876			
	HM3	0.898			
Price value (PV)	PV1	0.856	0.818	0.892	0.734
	PV2	0.883			
	PV3	0.831			
Habit (HA)	HA1	0.881	0.859	0.914	0.780
	HA2	0.904			
	HA3	0.864			
PTTA	PTTA1	0.837	0.843	0.905	0.761
	PTTA2	0.893			
	PTTA3	0.886			
PIET	PIET1	0.851	0.856	0.912	0.776
	PIET2	0.923			
	PIET3	0.868			
Behavioral intention (BI)	BI1	0.889	0.912	0.938	0.792
	BI2	0.906			
	BI3	0.914			
	BI4	0.849			
Use behavior (UB)	UB1	0.843	0.797	0.840	0.637
	UB2	0.714			
	UB3	0.831			

UB4 was removed due to low factor loading (0.587 < 0.70).

Discriminant validity was assessed using both the Fornell–Larcker criterion and the heterotrait-monotrait (HTMT) ratio, with results shown in Table 4. Under the Fornell–Larcker criterion, the square root of each construct’s AVE exceeded its correlations with all other constructs, confirming adequate discriminant validity. All HTMT ratios fell below the conservative threshold of 0.85, further confirming discriminant validity.

**Table 4:** Discriminant validity: HTMT ratios.

	PE	EE	SI	FC	HM	PV	HA	PTTA	PIET	BI	UB
PE	—										
EE	0.672	—									
SI	0.538	0.491	—								
FC	0.583	0.647	0.512	—							
HM	0.614	0.578	0.463	0.501	—						
PV	0.421	0.389	0.367	0.435	0.398	—					
HA	0.557	0.523	0.478	0.489	0.541	0.362	—				
PTTA	0.412	0.287	0.241	0.263	0.195	0.178	0.213	—			
PIET	0.296	0.253	0.219	0.367	0.174	0.156	0.198	0.683	—		
BI	0.701	0.658	0.547	0.591	0.623	0.412	0.614	0.374	0.341	—	
UB	0.489	0.467	0.398	0.521	0.443	0.356	0.537	0.298	0.267	0.631	—

Of the nine hypothesized paths, seven achieved statistical significance. Among UTAUT2’s original paths, performance expectancy had the strongest positive effect on behavioral intention (*β* = 0.213, *p* < 0.001, *f*^2^ = 0.058), followed by habit (*β* = 0.189, *p* < 0.001, *f*^2^ = 0.045), hedonic motivation (*β* = 0.174, *p* < 0.001, *f*^2^ = 0.039), and effort expectancy (*β* = 0.156, *p* = 0.002, *f*^2^ = 0.029). Social influence was significant but with a smaller effect size (*β* = 0.118, *p* = 0.007, *f*^2^ = 0.019). Facilitating conditions (*β* = 0.079, *p* = 0.093, *f*^2^ = 0.008) and price value (*β* = 0.062, *p* = 0.112, *f*^2^ = 0.006) did not reach significance, with near-negligible effect sizes. For the context-specific paths, PTTA had a significant negative effect on behavioral intention (*β* = −0.143, *p* < 0.001, *f*^2^ = 0.031), and PIET also reached significance though with a smaller effect size (*β* = −0.098, *p* = 0.030, *f*^2^ = 0.014). Behavioral intention significantly predicted use behavior with a large effect size (*β* = 0.631, *p* < 0.001, *f*^2^ = 0.662) (see Table 5).

**Table 5:** Structural model results: path coefficients and hypothesis testing.

Hypothesis	Path	*β*	SE	*t*-value	*p*-value	*f* ^2^	Result
H1	PE → BI	0.213	0.048	4.438	<0.001	0.058	Supported
H2	EE → BI	0.156	0.051	3.059	0.002	0.029	Supported
H3	SI → BI	0.118	0.044	2.682	0.007	0.019	Supported
H4	FC → BI	0.079	0.047	1.681	0.093	0.008	Not supported
H5	HM → BI	0.174	0.046	3.783	<0.001	0.039	Supported
H6	PV → BI	0.062	0.039	1.590	0.112	0.006	Not supported
H7	HA → BI	0.189	0.049	3.857	<0.001	0.045	Supported
H8	PTTA → BI	−0.143	0.042	3.405	<0.001	0.031	Supported
H9	PIET → BI	−0.098	0.045	2.178	0.030	0.014	Supported
—	BI → UB	0.631	0.037	17.054	<0.001	0.662	Supported

*f*^2^ effect sizes: 0.02 = small, 0.15 = medium, 0.35 = large.

Multi-group analysis results for moderating effects are presented in Table 6. Most moderating effects did not reach statistical significance, but three noteworthy significant effects emerged. Regarding age, habit’s positive effect on behavioral intention was significantly stronger among teachers under 35 than those 35 and above (Δ*β* = 0.112, *p* = 0.037), indicating that younger teachers’ technology use habits more strongly drive their adoption decisions. Regarding teaching experience, PIET’s negative effect on behavioral intention was significantly stronger among teachers with over 10 years of experience (Δ*β* = −0.138, *p* = 0.012), suggesting that accumulated experience reinforces teachers’ belief in the irreplaceability of embodied teaching. Regarding instrument category, PIET’s negative effect was significantly stronger among non-piano instrument teachers than piano teachers (Δ*β* = −0.121, *p* = 0.028), possibly related to the greater dependence on embodied guidance in string and wind teaching (e.g., bowing control, embouchure adjustment).

**Table 6:** Multi-group analysis results: moderating effects of age, teaching experience, and instrument category.

Path	Moderator	Group comparison	Δ*β*	*p*-value (permutation)
PE → BI	Age	≤35 vs. >35	0.067	0.218
EE → BI	Age	≤35 vs. >35	−0.089	0.146
HA → BI	Age	≤35 vs. >35	0.112	0.037*
PTTA → BI	Teaching experience	≤10 yrs. vs. >10 yrs	−0.094	0.081
PIET → BI	Teaching experience	≤10 yrs. vs. >10 yrs	−0.138	0.012*
HM → BI	Teaching experience	≤10 yrs. vs. >10 yrs	0.076	0.263
PTTA → BI	Instrument category	Piano vs. non-piano	−0.058	0.347
PIET → BI	Instrument category	Piano vs. non-piano	−0.121	0.028*
PE → BI	Instrument category	Piano vs. non-piano	0.043	0.489

*p < 0.05. Only selected paths with theoretical relevance are reported.

### 4.2 Phase 2: qualitative results

Based on Phase 1 quantitative results, 17 instrumental music teachers were selected from survey respondents using a maximum variation sampling strategy for semi-structured interviews. Interviewee profiles are presented in Table 1.

Reflexive thematic analysis of interview data yielded four core themes and their subthemes, as presented in Table 7.

**Table 7:** Thematic analysis: themes, subthemes, and representative quotes.

Theme	Subtheme	Representative quote
1. AI as a conditional efficiency tool	1a. Value in repetitive practice support	“AI-assisted practice software is genuinely helpful for intonation and rhythm training—students can use it for drilling fundamentals on their own after lessons, but that is only a small part of what teaching involves.” (T8)
	1b. Limitations in nuanced feedback	“The software can only tell you whether a note is in tune or a rhythm is correct, but music is not just about right and wrong—what matters more is how to express it.” (T3)
2. Artistry under perceived threat	2a. Standardization vs. individuality	“Every student has a different level of musical understanding. I tailor my guidance on how to interpret a piece based on each student’s personality and sensitivity—how could a machine do that?” (T12)
	2b. Erosion of aesthetic authority	“If students start treating the AI score as the definitive answer, then the teacher’s authority in aesthetic judgment is effectively hollowed out—that is my biggest concern.” (T9)
3. The irreplaceable body	3a. Physical contact and postural correction	“When teaching violin, I need to lift the student’s hand and adjust the angle of their shoulder—that is not something a screen can solve.” (T2)
	3b. Breath and embodied musicality	“In wind instrument teaching, breath control is the most essential element. I need the student to place their hand on my abdomen to feel how I breathe—AI simply cannot do this.” (T6)
	3c. Instrument-specific variation	“Piano teaching is relatively more amenable, but strings and winds demand such fine-grained physical precision that even the angle of a single small joint affects the tone.” (T14)
4. External enablers and barriers	4a. Institutional pressure and peer influence	“The school requires us to explore smart teaching and the leadership encourages adopting new technologies, but in practice nobody tells you how to use them in a one-on-one instrumental lesson.” (T11)
	4b. Training gaps and resource constraints	“It is not that I am unwilling to use it—I genuinely do not know how, and there is no training designed for instrumental teaching. All the tutorials online are geared toward academic subjects.” (T15)

Quotes were originally in Chinese and translated into English by the authors. Participant IDs correspond to Table 1.

Thematic analysis revealed that the attitude towards AI teaching tools by instrumental music teachers is marked by a conditional acceptance attitude. As shown in Theme 1, teachers position these technologies not as a replacement of their traditional methods but as complementary tools that enable them to conduct standardized and repetitive instruction such as pitch and rhythm exercises. With regard to dimensions of instruction concerning musical expressivity and performance interpretation, teachers viewed AI-generated feedback as oversimplified and reductive. Theme 2 exposes more serious professional fears concerning the possible damage conducted to teaching artistry; specifically, fear that AI-based tests may limit opportunities for personalized instruction, and also that AI-assisted evaluation by students may challenge the authority of instructors in matters of aesthetics. Theme 3 highlights the various ways in which teaching through the body cannot be replaced. Teachers give examples of the necessity for touch and space in instrumental teaching. This is shown by many practical situations, such as how to place the hands, breathing, and postures. In particular, irreplaceability is noticeable when teachers teach string and wind instruments. Theme 4 encompasses external factors that impact the adoption process: although there is institutional pressure, it does not lead to adoption because of the lack of guidelines and training for instructional purposes.

### 4.3 Integrated analysis

Systematic comparison of quantitative and qualitative results was achieved through a joint display table (Table 8), presenting relationships of convergence, complementarity, and contradiction.

**Table 8:** Joint display: integration of quantitative and qualitative findings.

Quantitative finding	Qualitative finding	Integration inference
H1 supported: PE → BI (*β* = 0.213***)	Theme 1a: teachers acknowledge AI’s value in repetitive practice tasks but frame it as conditional and limited	Convergent with nuance: PE is significant but bounded—teachers’ performance expectations are domain-specific, high for basic training and low for artistic dimensions
H2 supported: EE → BI (*β* = 0.156**)	Theme 4b: Teachers report lack of instrument-specific training and guidance as a major usability barrier	Complementary: EE’s significance is amplified by practical training gaps—perceived ease of use is not only about interface design but about pedagogical applicability
H3 supported: SI → BI (*β* = 0.118**)	Theme 4a: institutional pressure exists but lacks actionable guidance for one-on-one instrumental teaching contexts	Complementary: SI’s relatively weaker effect is explained by the disconnect between top-down encouragement and bottom-up practical support
H4 not supported: FC → BI (*β* = 0.079, n.s.)	Theme 4b: resource constraints are acknowledged but teachers prioritize pedagogical fit over infrastructural availability	Explanatory: FC’s non-significance is clarified—even when resources are available, teachers do not adopt without perceived pedagogical relevance
H5 supported: HM → BI (*β* = 0.174***)	Theme 1a: younger teachers express curiosity and enjoyment when exploring AI tools during basic training tasks	Convergent: intrinsic enjoyment drives adoption intention, especially among teachers who view AI as a novel supplement rather than a replacement
H6 not supported: PV → BI (*β* = 0.062, n.s.)	Not prominently discussed; cost is rarely mentioned as a concern	Convergent: cost is not a salient factor—most AI tools are free or institutionally funded, making PV irrelevant in this context
H7 supported: HA → BI (*β* = 0.189***)	Theme 1a: teachers already using AI for personal purposes find it easier to extend use to teaching	Convergent: habitual technology users transfer their usage patterns to educational contexts
H8 supported: PTTA → BI (*β* = −0.143***)	Theme 2a, 2b: deep concerns about standardization suppressing individuality and eroding aesthetic authority	Strongly convergent: qualitative data richly illustrates the mechanism behind PTTA’s negative effect—fear of losing artistic judgment and personalized guidance
H9 supported: PIET → BI (*β* = −0.098*)	Theme 3a, 3b, 3c: vivid accounts of physical contact, breath demonstration, and instrument-specific embodied teaching	Strongly convergent with elaboration: qualitative data explains why PIET is significant and why it varies across instruments—embodied teaching is more central in strings/winds than piano

The integrated analysis revealed core findings at three levels. In terms of the convergence category, the significance of performance expectancy, habit, hedonic motivation, and the context-specific factors (PTTA and PIET) is highly substantiated by qualitative results, where PTTA and PIET have been most explicitly explained from a mechanism standpoint through interviews. With respect to the complementarities, qualitative data provided insights that were impossible to achieve with quantitative data concerning effort expectancy and social influence. Effort expectancy refers to more than just interface use; it includes the educational applicability of the tools “pedagogical applicability” for instrumental learning. The lesser influence of social influence is because of the disjunction between top-down institutional encouragement and bottom-up practical guidance. At the explanatory level, qualitative data offered plausible explanations for the two non-significant paths: facilitating conditions’ non-significance arose not from insufficient infrastructure but from teachers prioritizing “pedagogical compatibility” over “resource availability”; price value’s non-significance reflected the reality that most current AI education tools are free or institutionally funded. To summarize the integration outcomes, the meta-inferences drawn from the joint display (Table 8) can be organized into three categories. Five paths (PE, HA, HM, PTTA, PIET) exhibited convergence, where the qualitative themes directly corroborated the direction and significance of the quantitative effects, with PTTA and PIET receiving the richest mechanistic elaboration through interview data. Two paths (EE, SI) exhibited complementarity, where the qualitative findings did not contradict the quantitative results but extended them by revealing dimensions—pedagogical applicability for EE and the encouragement-guidance gap for SI—that the survey items alone could not capture. The two non-significant paths (FC, PV) were clarified through explanatory integration, where interview data provided contextual reasons for the absence of statistical effects. No instances of outright divergence between the two data strands were identified.

## 5 Discussion

### 5.1 Discussion of main findings

In this paper, a thorough investigation of music teachers’ acceptance of AI teaching technology based on UTAUT2 model has been carried out. The findings from the combined analysis of quantitative and qualitative data present a more complicated perception of technology acceptance compared to what is known from previous research. Of all the paths in UTAUT2, only performance expectancy proved to be the most positively related to behavioral intentions (*β* = 0.213), in line with the literature on AI acceptance among science teachers (Al Darayseh, 2023). But there were also significant caveats in the form of qualitative data: the expectations of instrumental teachers regarding their own performances show notable domain specificity, placing much emphasis on the value of AI in standardized basic skills dimensions like pitch verification and rhythmic training, but expressing little faith in its application to higher-level teaching dimensions concerned with music expressivity and artistry. This conditional pattern of performance expectancy is not often seen in studies of technology acceptance in other subjects.

Habit was the second-strongest predictor (*β* = 0.189), and qualitative data revealed a clear transmission channel: teachers who had developed routine AI use in daily activities (e.g., smart assistants, voice recognition) were more inclined to extend these behaviors into teaching, consistent with findings on behavioral tendencies in generative AI acceptance (Alagöz Hamzaj, 2025). Hedonic motivation also had a significant positive effect (*β* = 0.174); interview accounts suggest that teachers who framed AI as supplementary rather than substitutive were more likely to experience intrinsic enjoyment, aligning with research on teachers’ curiosity toward technology innovations (Petrucco et al., 2024).

Despite the positive impact that social influence had on an individual’s behavior (*β* = 0.118), it was the path with the smallest effect size among all other significant paths. This is further explained by qualitative results, as technological promotion within institutions is common; however, encouragement from a higher authority does not motivate individual learning because there are no clear guidelines on how to do it. This finding makes a substantive contribution to the body of knowledge on moderating factors affecting teacher acceptance of technology in higher education (García-Murillo et al., 2023), noting that for social influence to work effectively, there needs to be not only the presence of external pressure but also supportive resources. Notably, both facilitating conditions and price value failed to attain statistical significance. Based on an integrated analysis, facilitating conditions are due to the fact that the teachers have been emphasizing pedagogical compatibility over resource availability, while price value is because most of the AI-based education applications are available for free. It is important to note that several statistically significant paths exhibited small effect sizes according to conventional benchmarks (Cohen, 2013). Social influence (*f*^2^ = 0.019) and PIET (*f*^2^ = 0.014) approached or fell slightly below the conventional small-effect threshold of 0.02, while effort expectancy (*f*^2^ = 0.029) and PTTA (*f*^2^ = 0.031) only slightly exceeded it. These values indicate that, although the relationships are statistically detectable given the sample size, their individual practical impact on behavioral intention is modest. This underscores the value of the qualitative phase in this study: while the effect sizes suggest limited standalone predictive power, the interview data reveal that these constructs operate as nuanced, context-dependent mechanisms whose significance may not be fully captured by variance-based metrics alone. Researchers and practitioners should therefore interpret these paths as theoretically meaningful but practically contingent, rather than as strong standalone drivers of adoption.

Key theoretical contributions made by this research are the detrimental effects that PTTA and PIET have on behavioral intentions. Out of these two variables, PTTA is shown to be significantly more harmful, having the largest negative impact on behavioral intentions (*β* = −0.143). The qualitative analysis suggests that both mechanisms are at play in instrumental teachers’ concerns about how the AI-driven, standardized system of assessment might limit the scope of personalized teaching and delegitimize teachers’ judgments regarding aesthetic criteria. The threat to artistry is a concern that extends beyond technophobia and involves the fundamental element of teacher professional identity: artistic judgment authority. While this element has been recognized in the discussion of the use of digital technologies in music education within schools, it has never been empirically validated among specific groups of teachers (Cheng, 2025b). Intervention research on piano teaching in a mixed approach has revealed that AI-based feedback can lead to positive results in terms of skill development and self-efficacy (Wang, 2025), but such impacts remain limited to technically standardizable components instead of the artistic aspects that are usually emphasized by instrumental teachers. The effectiveness of PTTA points to a contradiction between the efficiency of technology and that of teachers because the better AI performs technologically, the more teachers become wary of its potential encroachment on artistic assessment.

PIET revealed to have an adverse effect on behavioral intentions (*β* = −0.098). Even though the effect size may be small, this effect was statistically significant and well-supported qualitatively, suggesting that the irrelevance of embodied teachers’ functions plays the role of a considerable resistance mechanism. In multi-group analysis, the results were extended to show that the adverse impact of PIET is even greater for teachers who have been teaching for more than 10 years, suggesting that increased involvement strengthens the feeling of the indispensability of physical interaction. Across instrument categories, PIET’s negative effect was significantly stronger among string and wind teachers than piano teachers, closely aligning with interviewees’ vivid descriptions of teaching scenarios involving bowing control, embouchure adjustment, and breath demonstration. These findings engage with systematic examinations of responsible AI applications in education (Zhu H. et al., 2025)—while advancing AI in education, the differentiated needs of various disciplines for embodied interaction must be fully respected.

### 5.2 Implications for theory and practice

This study makes theoretical contributions at three levels. At the model extension level, proposing and testing PTTA and PIET extends UTAUT2’s applicability to the special educational context of instrumental teaching, which is highly dependent on embodied interaction and artistic judgment, responding to calls in systematic reviews for context-based variable extension of UTAUT2. At the disciplinary specificity level, this study reveals how instrumental teaching’s two core attributes—artistry and embodiment—systematically shape teachers’ distinctive technology acceptance patterns, providing an empirical foundation for understanding the theoretical role of embodiment and artistry in technology acceptance. These insights may extend to sports education, dance education, surgical training, and other fields similarly dependent on embodied guidance. At the methodological level, the reflexive thematic analysis adopted in this study followed the most important recent practice guidelines for this methodology (Braun and Clarke, 2022), using conceptual and design thinking to guide theme construction (Braun and Clarke, 2023), avoiding the common pitfall of reducing thematic analysis to mechanical coding, and providing a replicable exemplar of rigor in the qualitative phase of mixed-methods research.

The results provide actionable insights for three stakeholder groups. In terms of AI learning tools, the significant negative influence of PTTA highlights the importance of not designing AI learning tools in a way that undermines the teacher’s artistry through standardized answers but rather presents them as supplemental information tools used in making professional decisions. In terms of the PIET model, the significant positive impact along with its differentiation based on the category of the instrument suggests the necessity of distinguishing between different types of instruments in the development process. These findings about the relatively small effect of social influences and the qualitative result indicating “ample encouragement but insufficient guidance” demonstrate that the strategy used to promote technological use by educational administrators and policymakers should move from institutional promotion at the macro level to training and guidelines for integrating teaching across different subject areas. In terms of professional development for instrumental teachers, the mixed methods analysis approach provides a systematic framework of evidence for analyzing opportunities for integrating AI teaching (Creswell and Inoue, 2025). This is helpful for teachers to make better decisions regarding adopting the technology with a clear understanding of its limitations.

### 5.3 Limitations and future directions

There are various limitations associated with this study. Sampling issues have been noted since only instrumental music teachers from China participated in the study. This implies that any difference among the Chinese music education system, the definition of teachers’ roles, and technology use in other nations could affect the cross-cultural applicability of the results obtained herein. Regarding methodology, this research is characterized by cross-sectional data collection. Consequently, no dynamics within the changing attitudes of music teachers can be observed during the use of emerging technologies. Regarding data characteristics, the self-reporting surveys might suffer from social desirability bias. Regarding integration strategy, even though joint display tables provide an orderly method of analyzing both quantitative and qualitative findings (Zhou et al., 2024), the success of such integrations is still somewhat hindered by how well the themes fit the data across quantitative and qualitative analyses, providing room for further development within educational technology research with mixed methods (Peters and Fàbregues, 2024).

Following from these limitations, future research should take several directions. Cross-cultural comparisons could consider the differential value of PTTA and PIET in music education systems that are different from one another (e.g., European classical versus East Asian systems). Longitudinal studies offer the prospect of uncovering insights into the evolving attitudes towards technology adoption among instrumental teachers as they gain relevant experience using AI technologies. Specifically directed and in-depth analyses concentrating on specific types of instruments (e.g., strings or winds) may help to define the differences in internal processes inherent in the embodied dimension of teaching and their consequences on technology adoption. Intervention studies provide an opportunity for researchers to observe the impact of AI technology directly through their use in real teaching scenarios, thus providing evidence to move AI research from merely perceptions to practical effectiveness.

## 6 Conclusion

Within the theoretical perspective of an extended UTAUT2 framework and employing an explanatory sequential mixed-method approach to research, this study examines the adoption process of AI-based teaching technology among instrumental music teachers and the mechanisms involved. The findings from the analyses conducted using 352 survey results and 17 interview results reveal that there are positive correlations among performance expectancy, habit, hedonic motivation, effort expectancy, and social influence, as compared to behavioral intention. In this regard, facilitating conditions and price value were insignificant. Both PTTA and PIET are important negative determinants of behavioral intention, with their harmful impacts being more severe among teachers who have prior experience and for instruments that involve physical training, like strings and winds. The qualitative findings provided additional insight into the situation described in these numbers, revealing that the instrumental teachers’ adoption of AI technology was not a question of whether they adopted it or rejected it but rather of conditional adoption limited by domain and teacher expertise. While teachers recognize the usefulness of AI as a support system in skill development, they strongly assert that AI does not have the capacity to replace them in areas such as aesthetics, expression, and physical engagement. The results expand the application of the UTAUT2 theory framework in relation to the unique context of instrumental music education, where both elements play an important role, and provide designers a boundary definition: technology is supposed to enhance the skills of teachers, not replace them. With the increasing presence of AI in various aspects of education, maintaining a proper balance between technology and teaching skills will pose a formidable task not only in instrumental music education but also in other fields of education that entail human interaction.

## Data Availability

The raw data supporting the conclusions of this article will be made available by the authors, without undue reservation.

## References

[ref1] Acikgul K. Sad S. N. (2026). Educators’ acceptance and use of AI software: UTAUT2 model. SAGE Open 16:21582440251413449. doi: 10.1177/21582440251413449

[ref2] Acquilino A. Scavone G. (2022). Current state and future directions of technologies for music instrument pedagogy. Front. Psychol. 13:835609. doi: 10.3389/fpsyg.2022.835609, 35391969 PMC8981027

[ref3] Al Darayseh A. (2023). Acceptance of artificial intelligence in teaching science: science teachers’ perspective. Comput. Educ. Artifi. Intell. 4:100132. doi: 10.1016/j.caeai.2023.100132

[ref4] Alagöz Hamzaj Y. (2025). Generative AI acceptance among future educators: personality and behavioral insights. Educ. Inf. Technol. 30, 23165–23188. doi: 10.1007/s10639-025-13678-3

[ref5] Alves A. C. Nogueira M. (2024). Embodiment consciousness in music performance pedagogy. Empir. Musicolog. Rev. 19, 10–24. doi: 10.18061/emr.v19i1.9571

[ref6] Braun V. Clarke V. (2021a). One size fits all? What counts as quality practice in (reflexive) thematic analysis? Qual. Res. Psychol. 18, 328–352. doi: 10.1080/14780887.2020.1769238

[ref7] Braun V. Clarke V. (2021b). Thematic Analysis: A Practical Guide. Thousand Oaks, CA: SAGE.

[ref8] Braun V. Clarke V. (2022). Conceptual and design thinking for thematic analysis. Qual. Psychol. 9, 3–26. doi: 10.1037/qup0000196

[ref9] Braun V. Clarke V. (2023). Toward good practice in thematic analysis: avoiding common problems and be(com)ing a knowing researcher. Int. J. Trans. Health 24, 1–6. doi: 10.1080/26895269.2022.2129597, 36713144 PMC9879167

[ref10] Bremmer M. Nijs L. (2022) Embodied music pedagogy. A theoretical and practical account of the dynamic role of the body in music education. In: Buchborn T. Baets T. De Brunner G. Schmid S. (Eds.) Music is What People Do. European Perspectives on Music Education. Innsbruck: Helbling

[ref11] Caffaratti L. B. Longobardi C. Badenes-Ribera L. Marengo D. (2025). AI adoption among adolescents in education: extending the UTAUT2 with psychological and contextual factors. Front. Artif. Intell. 8:1614993. doi: 10.3389/frai.2025.1614993, 40988918 PMC12451006

[ref12] Chan C. K. Y. Tsi L. H. Y. (2024). Will generative AI replace teachers in higher education? A study of teacher and student perceptions. Stud. Educ. Eval. 83:101395. doi: 10.1016/j.stueduc.2024.101395

[ref13] Chen Q. Pan L. Liu Y. Sun X. Lee J. (2026). Exploring the mediating role of attitude toward use in GenAI adoption for pre-service music teacher: insights from the UTAUT2 framework. Front. Educ. 11:1793554. doi: 10.3389/feduc.2026.1793554

[ref14] Cheng L. (2025a). The impact of generative AI on school music education: challenges and recommendations. Arts Educ. Policy Rev. 126, 255–262. doi: 10.1080/10632913.2025.2451373

[ref15] Cheng L. (2025b). “The use of digital technology in school music education: artificial intelligence and emerging practices,” in The Sage Handbook of School Music Education, eds. Luis Aróstegui J. Christophersen C. Nichols J. Matsunobu K. (Thousand Oaks, CA: Sage Publications).

[ref16] Cohen J. (2013). Statistical Power Analysis for the Behavioral Sciences. Abingdon: Routledge.

[ref17] Creswell J. W. Creswell J. D. (2017). Research Design: Qualitative, Quantitative, and Mixed Methods Approaches. Thousand Oaks, CA: Sage Publications.

[ref18] Creswell J. W. Inoue M. (2025). A process for conducting mixed methods data analysis. J. Gen. Fam. Med. 26, 4–11. doi: 10.1002/jgf2.736, 39776872 PMC11702478

[ref19] Creswell J. W. Plano Clark V. L. (2023). “Revisiting mixed methods research designs twenty years later,” in Handbook of Mixed Methods Research Designs, (Thousand Oaks, CA: Sage Publications).

[ref20] Demir S. Uşak M. (2025). Analyzing the implementation of PLS-SEM in educational technology research: a review of the past 10 years. SAGE Open 15:21582440251345950. doi: 10.1177/21582440251345950

[ref21] Du W. Liang R.-Y. (2024). Teachers’ continued VR technology usage intention: an application of the UTAUT2 model. SAGE Open 14:21582440231220112. doi: 10.1177/21582440231220112

[ref22] Fetters M. D. Tajima C. (2022). Joint displays of integrated data collection in mixed methods research. Int J Qual Methods 21:16094069221104564. doi: 10.1177/16094069221104564

[ref23] García-Murillo G. Novoa-Hernández P. Serrano Rodríguez R. (2023). On the technological acceptance of Moodle by higher education faculty—a nationwide study based on UTAUT2. Behav. Sci. 13:419. doi: 10.3390/bs13050419, 37232655 PMC10215421

[ref24] Hair J. Alamer A. (2022). Partial least squares structural equation modeling (PLS-SEM) in second language and education research: guidelines using an applied example. Res. Methods Appl. Linguist. 1:100027. doi: 10.1016/j.rmal.2022.100027

[ref25] Hair J. F. Hult G. T. M. Ringle C. M. Sarstedt M. Danks N. P. Ray S. (2021). Partial Least Squares Structural Equation Modeling (PLS-SEM) Using R: A Workbook. Cham: Springer.

[ref26] He S. Ren Y. (2025). Exploring pre-service music teachers’ acceptance of generative artificial intelligence: a PLS-SEM-ANN approach. Front. Psychol. 16:1571279. doi: 10.3389/fpsyg.2025.1571279, 40657578 PMC12247848

[ref27] Li S. Timmers R. (2021). Teaching and learning of piano timbre through teacher–student interactions in lessons. Front. Psychol. 12:576056. doi: 10.3389/fpsyg.2021.576056, 34177678 PMC8222694

[ref28] Li P.-p. Wang B. (2024). Artificial intelligence in music education. Int. J. Hum. Comput. Interact. 40, 4183–4192. doi: 10.1080/10447318.2023.2209984

[ref29] Merchán Sánchez-Jara J. F. González Gutiérrez S. Cruz Rodríguez J. Syroyid Syroyid B. (2024). Artificial intelligence-assisted music education: a critical synthesis of challenges and opportunities. Educ. Sci. 14:1171. doi: 10.3390/educsci14111171

[ref30] Michałko A. Campo A. Nijs L. Leman M. Van Dyck E. (2022). Toward a meaningful technology for instrumental music education: teachers’ voice. Front. Educ. 7:1027042. doi: 10.3389/feduc.2022.1027042

[ref31] Peters M. Fàbregues S. (2024). Missed opportunities in mixed methods EdTech research? Visual joint display development as an analytical strategy for achieving integration in mixed methods studies. Educ. Technol. Res. Dev. 72, 2477–2497. doi: 10.1007/s11423-023-10234-z, 37359491 PMC10173938

[ref32] Petrucco C. Conte A. Favino F. (2024). Teachers’ perceptions on the introduction of generative AI in schools: a mixed-method study on the opinions of 1,223 teachers in the Veneto region, Italy. Educ. Sci. Soc. 2, 17–37. doi: 10.3280/ess2-2024oa18406

[ref33] Shata A. (2025). “Opting out of AI”: exploring perceptions, reasons, and concerns behind faculty resistance to generative AI. Front. Commun. 10:1614804. doi: 10.3389/fcomm.2025.1614804

[ref34] Shaw B. P. (2024). Artificial intelligence and assessment: three implications for music educators. Music. Educ. J. 111, 19–25. doi: 10.1177/00274321241296118

[ref35] Tamilmani K. Rana N. P. Dwivedi Y. K. (2021a). Consumer acceptance and use of information technology: a meta-analytic evaluation of UTAUT2. Inf. Syst. Front. 23, 987–1005. doi: 10.1007/s10796-020-10007-6

[ref36] Tamilmani K. Rana N. P. Wamba S. F. Dwivedi R. (2021b). The extended unified theory of acceptance and use of technology (UTAUT2): a systematic literature review and theory evaluation. Int. J. Inf. Manag. 57:102269. doi: 10.1016/j.ijinfomgt.2020.102269

[ref37] Venkatesh V. Thong J. Y. L. Xu X. (2012). Consumer acceptance and use of information technology: extending the unified theory of acceptance and use of technology. MIS Q. 36, 157–178. doi: 10.2307/41410412

[ref38] Wang S. (2025). Hybrid models of piano instruction: how combining traditional teaching methods with personalized AI feedback affects learners’ skill acquisition, self-efficacy, and academic locus of control. Educ. Inf. Technol. 30, 12967–12989. doi: 10.1007/s10639-025-13359-1

[ref39] Wu Q. (2025). The application of artificial intelligence in music education management: opportunities and challenges. J. Comput. Methods Sci. Eng. 25, 2836–2848. doi: 10.1177/14727978251322675

[ref40] Xu S. Chen P. Zhang G. (2024). Exploring Chinese university educators’ acceptance and intention to use AI tools: an application of the UTAUT2 model. SAGE Open 14:21582440241290013. doi: 10.1177/21582440241290013

[ref41] Xue L. Rashid A. M. Ouyang S. (2024). The unified theory of acceptance and use of technology (UTAUT) in higher education: a systematic review. SAGE Open 14:21582440241229570. doi: 10.1177/21582440241229570

[ref42] Yim I. H. Y. Wegerif R. (2024). Teachers' perceptions, attitudes, and acceptance of artificial intelligence (AI) educational learning tools: an exploratory study on AI literacy for young students. Fut. Educ. Res. 2, 318–345. doi: 10.1002/fer3.65

[ref43] Yu X. Ma N. Zheng L. Wang L. Wang K. (2023). Developments and applications of artificial intelligence in music education. Technologies 11:42. doi: 10.3390/technologies11020042

[ref44] Zhou Y. Zhou Y. Machtmes K. (2024). Mixed methods integration strategies used in education: a systematic review. Methodol. Innov. 17, 41–49. doi: 10.1177/20597991231217937

[ref45] Zhu W. Huang L. Zhou X. Li X. Shi G. Ying J. . (2025). Could AI ethical anxiety, perceived ethical risks and ethical awareness about AI influence university students’ use of generative AI products? An ethical perspective. Int. J. Hum. Comput. Interact. 41, 742–764. doi: 10.1080/10447318.2024.2323277

[ref46] Zhu H. Sun Y. Yang J. (2025). Towards responsible artificial intelligence in education: a systematic review on identifying and mitigating ethical risks. Hum. Soc. Sci. Commun. 12:1111. doi: 10.1057/s41599-025-05252-6

